# Prospective evaluation of plasma Epstein–Barr virus DNA clearance and fluorodeoxyglucose positron emission scan in assessing early response to chemotherapy in patients with advanced or recurrent nasopharyngeal carcinoma

**DOI:** 10.1038/s41416-018-0026-9

**Published:** 2018-03-20

**Authors:** Brigette Ma, Edwin P Hui, Ann King, Sing F Leung, Michael KM Kam, Frankie Mo, Leung Li, Ki Wang, Herbert Loong, Ashley Wong, Charles ML Chan, KC Allen Chan, SC Cesar Wong, YM Dennis Lo, Anthony TC Chan

**Affiliations:** 1 State Key Laboratory of Oncology in South China, Sir Y K Pao Centre for Cancer, Department of Clinical Oncology, Hong Kong Cancer Institute and Prince of Wales Hospital, The Chinese University of Hong Kong, Sha Tin, Hong Kong SAR; 2 Department of Diagnostic and Interventional Radiology, Prince of Wales Hospital, The Chinese University of Hong Kong, Sha Tin, Hong Kong SAR; 3 Department of Chemical Pathology, Prince of Wales Hospital, The Chinese University of Hong Kong, Sha Tin, Hong Kong SAR; 4 Faculty of Health and Social Sciences, Department of Health Technology and Informatics, Polytechnic University, Hung Hom, Hong Kong

**Keywords:** Head and neck cancer, Head and neck cancer

## Abstract

**Background:**

Plasma Epstein–Barr virus (pEBV) DNA and fluorodeoxyglucose positron emission (PET) reflect tumour burden in advanced NPC. This study hypothesised that a dual endpoint based on assessing pEBV DNA clearance and PET response could predict early drug response.

**Methods:**

Eligible patients underwent a computed tomography (CT) scan and dual PET-CT at baseline, a PET-CT at 4 weeks, and then a CT scan at 10 weeks after starting palliative or induction chemotherapy. Plasma EBV DNA clearance was determined.

**Results:**

Fifty-eight out of 70 enrolled patients completed all imaging and 50/58 had falling pEBV DNA level, which allowed calculation of the clearance. At a median follow-up of 29.1 months, the dual endpoint (pEBV DNA clearance ≤ 10 days and > 50% drop in sum of SUVmax of target lesions) was an independent indicator of overall survival (hazard ratio (HR) = 0.135, 95% CI = 0.039 to 0.466, *p* = 0.0015) and progression-free survival (HR = 0.136, 95% CI = 0.048 to 0.385, *p* = 0002). This dual endpoint could predict subsequent response by Response Evaluation Criteria In Solid Tumours (RECIST) criteria at 10 weeks after chemotherapy.

**Conclusions:**

Early PET-CT response and pEBV DNA clearance could predict survival and subsequent response. This dual endpoint is an innovative tool for assessing early drug response.

## Introduction

The endemic form of non-keratinising nasopharyngeal carcinoma (NPC) is the prototypical ‘chemo-sensitive’ tumour, which is associated with a high objective response rate as based on conventional criteria such as the Response Evaluation Criteria In Solid Tumours (RECISTs) following chemotherapy.^1^ Despite reports of high response rates to platinum-based chemotherapy in the palliative setting, the median time to progression is at best around 7.3 to 10 months.^1^ Similarly, the high response rates reported in phase II trials of induction chemotherapy has been translated into overall survival (OS) benefit in some but not all randomised studies in stage III to IVb NPC.^2–5,6^ These observations may suggest that assessing drug response based on changes in tumour dimensions may not reliably predict survival in advanced NPC.

This study investigated an innovative approach of assessing early drug response by measuring both tumour metabolic activity and the plasma clearance of cell-free Epstein–Barr virus (pEBV) DNA following chemotherapy in patients with advanced NPC. The quantitation of pEBV DNA has been shown to reflect tumour burden and predict survival in patients with advanced NPC undergoing radiotherapy (RT) or chemotherapy.^7,8^ However, the use of fluorodeoxyglucose (18F-FDG) positron emission tomography (PET) in assessing early response to chemotherapy has not been well established in NPC, unlike in other solid tumours.^9–11^ Therefore, the main purpose of this study was to evaluate the prognostic significance of a composite endpoint based on assessing both PET response and pEBV DNA clearance at 4 weeks after starting chemotherapy for advanced NPC (referred to as the ‘dual endpoint’ thereafter). This study also tested the hypothesis that the dual endpoint could be more accurate than testing either modality alone in predicting subsequent response by RECIST criteria using a contrast-enhanced computed tomography (CT) scan at 10 weeks after starting chemotherapy. This study has been approved by the Joint Chinese University of Hong Kong—New Territories East Cluster Clinical Research Ethics Committee. All participants had given written informed consent prior to enrollment.

## Patients and methods

### Patient enrollment

All eligible patients were aged 18 years or above with an Eastern Cooperative Group (ECOG) performance status of 0 to 2. Patients were eligible for the study if they could give written informed consent and were deemed medically fit to undergo palliative chemotherapy for metastatic or recurrent NPC, or induction chemotherapy for stage III to IVB NPC before chemo-RT (CRT). The patients had to have measurable disease by RECIST criteria (version 1.1) and the choice of chemotherapy was determined at the physician’s discretion based on institutional practice.^1^

### Study procedure and quantitation of plasma EBV DNA clearance

The study procedures were performed at three time points. At baseline, all patients had a whole-body dual PET and plain CT (PET-CT), and a contrast-enhanced CT from the brain to mid-thigh, a whole-body PET with plain CT (without contrast) at 4 weeks (with a window of around 7 days) after starting chemotherapy and then a contrast-enhanced CT scan from the nasopharynx to pelvis at 10 weeks (window of around 7 days) after starting chemotherapy. Blood samples (5 ml) were obtained before treatment and then weekly for 4 consecutive weeks for computing the EBV DNA clearance. pEBV DNA analysis was performed using reverse transcriptase-PCR and its clearance (half-life in days) calculated as described by Lo et al.^8^

### Radiological examinations

The dual PET-CT (plain) were performed with the Philips GEMINI GXL PET-CT Imaging System (Phillips Medical Systems, The Netherlands) as previously described.^12^ The contrast-enhanced CT scan was performed on a helical CT scanner (VCT, General Electric Medical Systems, Milwaukee, WI). The PET images (with and without attenuation correction), CT images, and the dual PET-CT fusion images were interpreted in parallel.^12^ Visual and semi-quantitative assessment of the regions of interest (ROIs) was made by measuring the maximum standardised uptake value (SUVmax) normalised to injected activity and the patient’s body weight. ROIs with a SUVmax value of > 2.5 were regarded as pathological and a maximum of 10 target lesions were selected per patient per scan, with a maximum of 5 lesions per organ. There is currently no consensus over the optimal number of target lesions, which should be assessed, and the European Organisation for Research and Treatment of Cancer (EORTC) criteria does not specify the number of target lesions to be selected per scan.^11^ In the studies of PET as a tool for assessing response to treatment in patients with metastatic colorectal cancer, the number of detectable lesions which were selected for PET assessment have ranged between three or more lesions (i.e., some studies used all the available lesions).^13–15^ As such data are lacking in NPC, the maximum number of target lesions selectable for assessment (10 lesions) was thus arbitrarily chosen in this study, and the same lesions selected at baseline were assessed during the follow-up scans. The RECIST criteria (version 1.1) was used in this study.

### Data analysis and study endpoints

This study hypothesised that a composite endpoint (the ‘dual endpoint’) based on measuring the pEBV DNA clearance and PET response could predict early response to chemotherapy. The primary endpoint of this study was to determine if this dual-endpoint at 4 weeks after starting chemotherapy could predict OS or progression-free survival (PFS). The secondary endpoint was to test the hypothesis that this dual endpoint is more accurate than either modality alone, in predicting the subsequent RECIST response determined at 10 weeks after starting chemotherapy.

It remains unclear what constitutes the most clinically relevant percentage decline in the sum of SUVmax of target lesions during the first 4 weeks of chemotherapy. The EORTC criteria defined PET response as a decline in the SUVmax of a target lesion(s) of over 25% following more than one cycle of chemotherapy, whereas the PET Response Criteria in Solid Tumours recommended a larger decline of 30%.^9,11^ Moreover, higher thresholds of over 50% have been found to be more clinically relevant in specific cancers.^9^ In the current study, analysis with a receiver operating characteristic curve did not reveal an optimal cutoff value; therefore, a range of SUVmax thresholds (30%, 40%, and 50% drop) were analysed using a multivariate analysis as explained below.

The respective median half-lives of pEBV DNA following salvage surgery, RT, and chemotherapy have been reported to be 139 min, 3.8 days, and 4 days, respectively.^7,8,16^ Wang et al.^7^ found that patients with pEBV DNA clearance of ≤ 8 days had longer OS following palliative chemotherapy for NPC. The median pEBV DNA clearance (half-life) was 10 days in the current study. Therefore, given the findings of Wang et al.,^7^ this study tested thresholds of ≤ 8, 10, or 15 days in a multivariate analysis.

For the survival endpoints, OS was defined as the time from study registration to death due to any cause and PFS was defined as the time from study registration to the first event of cancer progression or death from any causes. Survival rates and curves were estimated via the Kaplan–Meier method and compared using the log-rank test. Hazard ratios (HRs) with 95% confidence intervals (CIs) were estimated with the Cox Proportional Hazard Model and were adjusted for age, gender, disease stage, and Eastern Cooperative Oncology Group (ECOG) performance status. The optimal definition of the dual endpoint was derived as follow. Initially, prognostic variables were entered in an univariate analysis, which showed a significant association between survival and two thresholds of pEBV DNA clearance (‘ ≤ 10’ and ‘ ≤ 15 days’), and a PET response threshold of ‘> 50% drop in sum of SUVmax of target lesions.’ Subsequently, two definitions of the dual endpoint were re-entered as variables into the Hazard Model, with pEBV DNA clearance and PET response (% drop in sum of SUVmax of target lesions) being defined respectively as: (1) ‘≤ 10 days and ;> 50% drop,’ and (2) ‘≤ 15 days and > 50% drop (Supplemental Tables 1 and 2). The area under the curve ‘AUC’ method and Harrell’s c-index were used to select the optimal thresholds of the dual endpoint. The c-index and 95% conference intervals were calculated using the SAS macro program and the different thresholds were compared using the bootstrap method with 1000 replications.

This variable was then correlated with RECIST response in a logistic regression and the accuracy was calculated using the AUC operator characteristic curve method.

### Sample size calculation

The sample size of this study was calculated based on the hypothesis that the dual endpoint could distinguish between the median survival of responders and non-responders following chemotherapy. As there is no published report comparing the median OS or PFS of responders and non-responders with chemotherapy in advanced NPC, it was postulated that the HR for death (or progression) could be approximately between 2.0 and 2.5, assuming that survival follows an exponential distribution. Thus, a total of 68 patients would be recruited with a two-sided ɑ-level of 0.05 and a power of 0.8.

## Results

A total of 70 patients were recruited according to the planned sample size; however, 12 patients withdrew from the study after having the baseline PET-CT scan and blood tests—2 patients were found to have brain metastases on the PET-CT, 1 patient deteriorated after the first cycle of chemotherapy, and the rest withdrew due to personal reasons. Therefore, a total of 58 patients completed all protocol-related imaging and of these patients, and pEBV DNA clearance could be determined from 50 patients who achieved a falling level of pEBV DNA at 4 weeks after starting chemotherapy. Details on the demographics are summarised in Table 1. Of the 58 evaluable patients, 33 patients had recurrent or metastatic NPC, of whom 31 (93.9%) were undergoing first-line and 2 (6.1%) patients were undergoing second-line palliative chemotherapy at the time of enrollment, respectively. In the induction subgroup (*n* = 25), they were all treatment-naive at the time of enrollment and were treated with platinum-based chemotherapy followed by concurrent CRT. There was no statistical difference in the number of events (death or progression) among patients with one site versus more than one site of metastases. The majority of the palliative subgroup (*n* = 24, 75%) and the induction subgroup (*n* = 20, 80%) received platinum and gemcitabine, and the rest had platinum-taxanes or 5-fluorouracil.

**Table 1:** Patient characteristics and treatment response

Clinical characteristics	Evaluable patients, *n* = 58
Age: mean (SD) years	50.0 (± 9.2)
Gender	
Male	48 (82.8%)
Female	10 (17.2%)
ECOG	
0	25 (43.1%)
1 to 2	33 (56.9%) – 4 patients had ECOG 2
Disease stage	
Metastatic/ recurrent	33 (56.9%)
Locoregionally advanced	25 (43.1%)
Plasma EBV DNA	
Baseline (median copies/ml, range)	23,838 (31–1,924,668)
Clearance: mean (days, SD)	24.6 (± 48.6) days
Clearance: median (days, range)	10.0 (2.6–322.1) days
Clearance: ≤ 10 days (patients)	24 (52.0%)^a^
Clearance: > 10 days (patients)	26 (48.0%)^a^
PET response at 4 weeks	
SUVmax drop: > 50%	22 (37.9%)
SUVmax drop: ≤ 50%	36 (62.1%)
Dual response: (pEBV DNA and % drop sum of SUVmax of target lesions)	
≤ 10 days and > 50% drop: yes	15 (30%)
≤ 10 days and > 50% drop: no	35 (70%)
RECIST (1.1) response at 10 weeks	
CR	0
PR	32 (55.2%)
SD	22 (37.9%)
PD	4 (6.9%)
Baseline SUVmax and number of lesions selected	Median and range
Pre-study SUVmax of target lesions	34.25 (2.5–123.6).
Number of lesion selected (RECIST)	5.5 lesions (1–9)

*ECOG*, Eastern Cooperative Oncology Group performance status.

^a^Plasma EBV DNA clearance/half-life could be determined from 50 patients who demonstrated a falling trend at 4 weeks after chemotherapy

Using the AUC method and the Harrell’s c-index, the Akaike Information Criterion of the threshold ‘pEBV DNA CL ≤ 10 days and > 50% drop in sum of SUVmax’ (150.97) was found to be lower than the other threshold. There was no difference in c-index values between the different thresholds of dual endpoints used (*p* = 0.7822) Therefore, the dual endpoint ‘pEBV DNA CL ≤ 10 days and > 50% drop in sum of SUVmax’ was deemed the most optimal threshold in this study.

**Fig. 1:**
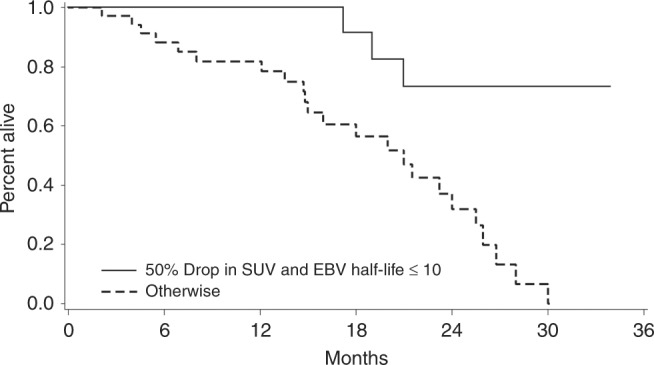
Overall survival (OS) curves of responders (top curve, > 50% drop in sum of SUVmax of target lesions and plasma EBV DNA clearance of ≤ 10 days, 1-year OS 100%) versus non-responders (bottom curve, ≤ 50% drop in sum of SUVmax and plasma EBV DNA clearance of > 10 days, 1-year OS 81.9%) based on the dual endpoint (log-rank, *p* = 0.0003). For the evaluable cohort (*n* = 58), the number of death and progression are 30 and 35

Regarding OS for the evaluable cohort (*n* = 58), the median OS was 25.5 months (95% CI = 19.8 to 30.2 months) at a median follow-up of 29.1 months (95% CI = 23.2 to 33.9 months). The result of the univariate analysis is shown in Supplemental Table 1. In the multivariate analysis, only the dual endpoint (defined as pEBV DNA clearance ≤ 10 days and > 50% drop in sum of SUVmax) was the only independent predictor of OS (HR 0.135, 95% CI = 0.039 to 0.466, *p* = 0.0015). The median OS for patients who achieved this dual endpoint at 4 weeks after starting chemotherapy was longer than those who could not (median OS = not reached (95% CI = 19.8months to ‘not reached’) versus 21.5 months (95% CI = 14.9 to 25.6 months), log-rank *p* = 0.0003, see Fig. 1).

**Fig. 2:**
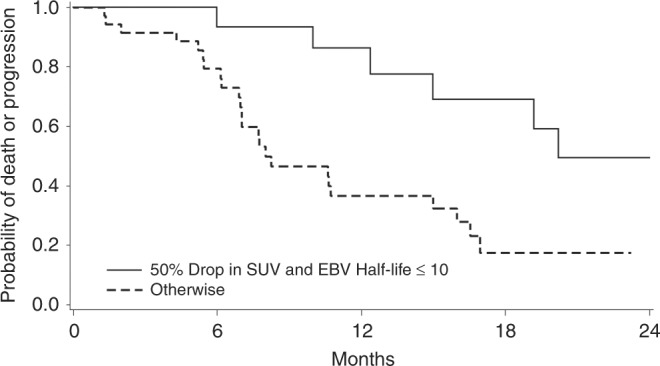
Progression-free survival (PFS) curves of responders (top curve, > 50% drop in sum of SUVmax and plasma EBV DNA clearance of ≤ 10 days, 1-year PFS rate 86.2%) versus non-responders (bottom curve, ≤ 50% drop in SUVmax and plasma EBV DNA clearance of > 10 days, 1-year PFS 36.5%) based on the dual endpoint (log-rank *p* = 0.0047)

Concerning PFS, the median PFS of the evaluable cohort (*n* = 58) was 12.4 months (95% CI = 8.7 to 19.3 months). Result of the univariate analysis is summarised in Supplemental Table 2. Multivariate analysis showed that the dual endpoint (defined as pEBV DNA ≤ 10 days and > 50% drop in sum of SUVmax, HR = 0.136, 95% CI = 0.048 to 0.385, *p* = 0.0002) and the presence of distant metastasis (HR = 9.13, 95% CI = 3.63 to 22.96, *p* < 0.0001) were independent predictor of PFS. The median PFS of responders versus non-responders according to the dual endpoint were 20.8 months (95% CI = 12.4 to ‘not reached’) and 7.9 months (95% CI = 7.0 to 15.5 months, log-rank *p* = 0.0047, Fig. 2), respectively.

Following the establishment of the dual endpoint (pEBV DNA ≤ 10 days and > 50% drop in sum of SUVmax) as an independent prognostic factor, this study further examined whether this endpoint could predict RECIST response at 10 weeks after chemotherapy more accurately than if either PET response (> 50% drop in sum of SUVmax) or pEBV DNA clearance (≤ 10 days) was used alone. Result of the univariate analysis is summarised in Supplemental Table 3. The dual endpoint was the only independent predictor of RECIST response in the multivariate analysis, with an odds ratio of 16.62 (95% CI = 1.966 to 140.551, *p* = 0.0099). The dual endpoint has highest specificity (95%) compared with PET response (90%) and pEBV DNA clearance alone (75%). However, PET response alone has the highest accuracy (AUC 75%, 95% CI = 61.3 to 88.7%) followed by the dual endpoint (AUC 70.8%, 95% CI = 56.6 to 85.1%) and pEBV DNA clearance (AUC 69.2%, 95% CI = 54.1 to 84.2%).

This study initially allowed only patients who were planned for palliative chemotherapy to be enrolled, but the protocol was amended later to include those who were eligible for induction chemotherapy. A post-hoc subgroup analysis on patients who received induction versus palliative chemotherapy was performed to adjust for the different prognosis between these subgroups. Details of the univariate analyses for the palliative and induction subgroups are outlined in the Supplemental Tables 4 to 7. In the induction subgroup (*n* = 25, Supplemental Tables 6 and 7) following a median follow-up of 25.9 months (95% CI = 16.9 to 29.1 months), none of the prognostic factors showed statistical association with survival or RECIST response. For the palliative subgroup (*n* = 33, Supplemental Table 4 to 5) following a median follow-up of 36.7 months (95% CI = 29.2 to 51.9 months), a multivariate analysis showed that the dual endpoint was the only independent predictor of OS (HR = 0.235, 95% CI = 0.068 to 0.817, *p* = 0.0228). The association with PFS was significant in the univariate but not the multivariate analysis (Supplemental Table 5), probably because this subgroup was underpowered. Interestingly, when the log-rank test was used to compare the PFS curves in the metastatic subgroup (*n* = 28), patients who achieved the dual endpoint had longer median PFS (15.3 months) than those who did not (median 7 months, *p* = 0.0029).

## Discussion

This study showed that assessment of early drug response based on evaluating both changes in metabolic and plasma clearance of cell-free EBV DNA, has both prognostic and predictive significance in patients undergoing chemotherapy for advanced NPC. Patients who achieved an early response based on this criterion at 4 weeks after starting chemotherapy were more likely to experience longer survival than those who did not. This composite endpoint has higher specificity in predicting the subsequent RECIST response at 10 weeks after chemotherapy than when either components of the composite endpoint were assessed alone. Although PET response alone has the highest overall accuracy than the dual endpoint in predicting RECIST response, there is significant overlap in the CIs between the two endpoints.

The combined use of functional imaging and circulating biomarker in assessing early response to anti-cancer therapy is a novel concept in oncology. The reasoning behind this approach is that changes in tumour dimension may not be apparent after 1 or 2 cycles of chemotherapy; therefore, the use of modalities that do not rely on measuring dimensional changes may be better at detecting early response. The authors have recently reported a prospective study, which found that the combined use of circulating tumour cells and PET-CT response was prognostic when used to assess early response to chemotherapy in metastatic colorectal cancer.^15^ Considering the result in the current study, this new approach may be valid across different cancers.

There is now substantial literature that supports the utility of FDG-PET as an early indicator of response to chemotherapy.^9,11^ However, there is no consensus on the most optimal PET metrics for measuring metabolic response. A variety of parameters have been used, such as measuring changes in SUVmax-based parameters (e.g., total SUVmax of all target lesions, SUVpeak, SUVmean, standard uptake lean body mass, and the highest SUVmax in a single lesion), and volumetric-based parameters (e.g., total lesion glycolysis (TLG) and metabolic tumour volume (MTV)).^9,11,17,18^ Irrespective of the PET metrics used, a consistent prognostic significance is found in the literature. For example, in NPC, a meta-analysis of 14 studies found that a single PET performed before RT has prognostic significance irrespective of the kind of metrics (SUVmax, MTV, or TLG) used to assess metabolic activity of the primary tumours.^18^ Several studies have reported the superiority of PET at 3 months after completing CRT in detecting residual tumour compared with other imaging in stage IVA to IVB NPC.^19–21^ However, there is a lack of prospective studies on PET as an indicator of early response to chemotherapy using PET parameters. Yen et al.^10^ found that patients with locally advanced NPC, who achieved a pre-defined degree of tumour down-staging on a PET scan within 7 to 14 days of starting induction chemotherapy, experienced longer recurrence-free survival and OS. However, this study did not use PET parameters to define response. In another small series of 20 patients with locally advanced NPC, ^22^ a decline of 70% in the sum of SUVmax of the target lesions following two cycles of induction chemotherapy was found to predict MRI response. The current study is unique in showing that PET response was significantly correlated with both survival (OS) and RECIST response in NPC in the univariate analyses, which is consistent in other solid cancers.^9,11^

Our previous work on the kinetics of pEBV DNA following RT or surgery supports the concept that pEBV DNA is an accurate marker of tumour burden in NPC.^8,16,23^ In the report by Wang et al.^7^ on patients undergoing cisplatin-epirubicin-mitomycin for recurrent NPC, plasma clearance thresholds of 4 to 8 days were associated with OS. In the current study, the median half-life was 10 days and thus longer than that reported by Wang et al.^7^ This could be due to inter-assay variability, differences in patient selection, or the choice of chemotherapy when compared with the current study. Despite the differences, the current study showed that pEBV DNA clearance of 8 days, 10 days, and 15 days all showed either a statistical trend or significant association with OS in the univariate analysis (Supplemental Table 1). A potential limitation of this study is the inclusion of the induction subgroup of patients who have better prognosis than the palliative subgroup. This may potential confound the association with OS and PFS in the combined analysis of all enrolled subjects. However, a post-hoc subgroup analysis found that the dual endpoint remained significantly associated with OS in the palliative subgroup, while a nonsignificant trend was observed with PFS. A post-hoc re-calculation of the study power was also conducted, given that 58 patients completed the protocol-related imaging and of whom 50 patients had falling EBV DNA levels which enabled the determination of the EBV DNA clearance. If two-sided *ɑ* of 0.05 was used, then the power would be 66.7% when 50 patients were for all study endpoints. Conversely, if one-sided *ɑ* of 0.05 was used then the power would be 77.2%. This is acceptable given that this study was intended to be a pilot study.

This prospective study has demonstrated the prognostic and predictive significance of combining PET response and pEBV DNA clearance in assessing early response to chemotherapy in advanced NPC. This dual endpoint has the potential of creating a window of opportunity to modify treatment decisions at an early time point, e.g., in identifying the patients who are more likely to benefit from induction chemotherapy prior to CRT, in light of the results of recently published randomised studies on this approach.^3,5,24,6^ In the palliative setting, this tool may provide an early signal of activity in the evaluation of novel agents in NPC. However, the current pilot study is underpowered for the induction cohort, therefore validation in larger cohorts is warranted.

## Electronic supplementary material

Supplementary Table 1

Supplementary Table 2

Supplementary Table 3

Supplementary Table 4

Supplementary Table 5

Supplementary Table 6

Supplementary Table 7
