# Supplementary material for: Prospective evaluation of plasma Epstein–Barr virus DNA clearance and fluorodeoxyglucose positron emission scan in assessing early response to chemotherapy in patients with advanced or recurrent nasopharyngeal carcinoma
Source: Br J Cancer. 2018 Mar 20;118(8):1051–5. doi: 10.1038/s41416-018-0026-9 (PMC5931094; doi:10.1038/s41416-018-0026-9)
Supplement: Supplementary file 1 — Supplementary Table 1 [file 41416_2018_26_MOESM1_ESM.docx]

**Supplemental Table 1: Overall survival - all patients (univariate analysis)**

| **Variable name** | **N** | **P-value** | **Hazard Ratio** | **95% C.I.** |
| --- | --- | --- | --- | --- |
| Advanced age | 58 | 0.0815 | 1.038 | 0.995-1.082 |
| Male gender | 58 | 0.3028 | 1.876 | 0.567-6.213 |
| ECOG performance (0 v.s. 1-2) | 58 | 0.3621 | 1.402 | 0.678-2.902 |
| Metastatic or non-metastatic | 58 | **0.0146** | 2.891 | 1.233-6.775 |
| >30% drop in sum of SUVmax | 58 | 0.1760 | 0.606 | 0.294-1.252 |
| >40% drop in sum of SUVmax | 58 | 0.1003 | 0.546 | 0.266-1.124 |
| >50% drop in sum of SUVmax | 58 | **0.0330** | 0.411 | 0.182-0.931 |
| RECIST (version 1.1) response | 58 | 0.1580 | 0.590 | 0.283-1.227 |
| pEBV DNA CL < 8 days | 54 | 0.0643 | 0.394 | 0.147-1.057 |
| pEBV DNA CL < 10 days | 54 | **0.0318** | 0.407 | 0.179-0.925 |
| pEBV DNA CL < 15 days | 54 | **0.0285** | 0.417 | 0.190-0.912 |
| pEBV DNA CL <10 & >50% drop in sum of SUVmax | 50 | **0.0015** | 0.135 | 0.039-0.466 |
| pEBV DNA CL <15 & >50% drop in sum of SUVmax | 50 | **0.0015** | 0.169 | 0.056-0.505 |

(**Legend**: ECOG PS = eastern cooperative group performance status, SUVmax = maximal standard uptake value, CL = clearance, CI = confidence interval, pEBV DNA = plasma Epstein Barr virus DNA)
