# Supplementary material for: Prospective evaluation of plasma Epstein–Barr virus DNA clearance and fluorodeoxyglucose positron emission scan in assessing early response to chemotherapy in patients with advanced or recurrent nasopharyngeal carcinoma
Source: Br J Cancer. 2018 Mar 20;118(8):1051–5. doi: 10.1038/s41416-018-0026-9 (PMC5931094; doi:10.1038/s41416-018-0026-9)
Supplement: Supplementary file 2 — Supplementary Table 2 [file 41416_2018_26_MOESM2_ESM.docx]

**Supplemental Table 2: Progression-free survival - all patients (univariate analysis)**

| **Variable name** | **N** | **P-value** | **Hazard Ratio** | **95% C.I.** |
| --- | --- | --- | --- | --- |
| Advanced age | 58 | 0.1553 | 1.028 | 0.990-1.067 |
| Male gender | 58 | 0.6996 | 1.205 | 0.467-3.112 |
| ECOG performance (0 v.s. 1-2) | 58 | 0.6358 | 1.170 | 0.610-2.245 |
| Metastatic or non-metastatic | 58 | **<0.0001** | 5.828 | 2.603-13.052 |
| >30% drop in sum of SUVmax | 58 | 0.3740 | 0.737 | 0.377-1.443 |
| >40% drop in sum of SUVmax | 58 | 0.2336 | 0.671 | 0.348-1.294 |
| >50% drop in sum of SUVmax | 58 | 0.1263 | 0.583 | 0.292-1.164 |
| RECIST 1.1 response | 58 | 0.2389 | 0.674 | 0.350-1.299 |
| pEBV DNA CL < 8 days | 54 | **0.0188** | 0.336 | 0.135-0.835 |
| pEBV DNA CL < 10 days | 54 | **0.0078** | 0.354 | 0.165-0.760 |
| pEBV DNA CL < 15 days | 54 | **0.0166** | 0.400 | 0.189-0.847 |
| pEBV DNA CL <10 & >50% drop in sum of SUVmax | 50 | **0.0075** | 0.281 | 0.111-0.712 |
| pEBV DNA CL <15 & >50% drop in sum of SUVmax | 50 | **0.0313** | 0.398 | 0.172-0.921 |

(**Legend**: ECOG PS = eastern cooperative group performance status, SUVmax = maximal standard uptake value, CL = clearance, CI = confidence interval, pEBV DNA = plasma Epstein Barr virus DNA)
