# Supplementary material for: Prospective evaluation of plasma Epstein–Barr virus DNA clearance and fluorodeoxyglucose positron emission scan in assessing early response to chemotherapy in patients with advanced or recurrent nasopharyngeal carcinoma
Source: Br J Cancer. 2018 Mar 20;118(8):1051–5. doi: 10.1038/s41416-018-0026-9 (PMC5931094; doi:10.1038/s41416-018-0026-9)
Supplement: Supplementary file 3 — Supplementary Table 3 [file 41416_2018_26_MOESM3_ESM.docx]

**Supplemental table 3: RECIST response (univariate analysis, logistic regression)**

| **Variable name** | **N** | **P-value** | **Odd Ratio (OR)** | **95% C.I. for OR** |
| --- | --- | --- | --- | --- |
| Advanced age | 58 | 0.8845 | 1.004 | 0.949-1.063 |
| Male gender | 58 | 0.0995 | 0.250 | 0.048-1.301 |
| Grouped ECOG (0 v.s. 1-2) | 58 | 0.2416 | 0.529 | 0.183-1.535 |
| Metastatic or non-metastatic | 58 | 0.5205 | 0.708 | 0.247-2.028 |
| >50% drop in sum of SUVmax | 58 | **0.0026** | 7.071 | 1.978-25.276 |
| pEBV DNA CL < 10 days | 50 | **0.0102** | 5.182 | 1.477-18.182 |
| Dual Endpoint (pEBV DNA CL <10 & >50% drop in sum of SUVmax)  EBVDNA half-life <=10 & 50% drop in SUV | 50 | **0.0099** | 16.622 | 1.966-140.551 |

(**Legend**: ECOG PS = eastern cooperative group performance status, SUVmax = maximal standard uptake value, CL = clearance, CI = confidence interval, pEBV DNA = plasma Epstein Barr virus DNA)
