# Supplementary material for: Prospective evaluation of plasma Epstein–Barr virus DNA clearance and fluorodeoxyglucose positron emission scan in assessing early response to chemotherapy in patients with advanced or recurrent nasopharyngeal carcinoma
Source: Br J Cancer. 2018 Mar 20;118(8):1051–5. doi: 10.1038/s41416-018-0026-9 (PMC5931094; doi:10.1038/s41416-018-0026-9)
Supplement: Supplementary file 4 — Supplementary Table 4 [file 41416_2018_26_MOESM4_ESM.docx]

**Supplemental Table 4: Overall survival – palliative subgroup (univariate analysis)**

| **Variable name** | **N** | **P-value** | **Hazard Ratio** | **95% C.I.** |
| --- | --- | --- | --- | --- |
| Advanced age | 33 | 0.0157 | 1.071 | 1.013-1.132 |
| Male gender | 33 | 0.6166 | 1.454 | 0.336-6.302 |
| ECOG performance (0 v.s. 1-2) | 33 | 0.3186 | 1.532 | 0.662-3.543 |
| >30% drop in sum of SUVmax | 33 | 0.2285 | 0.602 | 0.264-1.375 |
| >40% drop in sum of SUVmax | 33 | 0.1953 | 0.578 | 0.252-1.325 |
| >50% drop in sum of SUVmax | 33 | 0.1034 | 0.475 | 0.194-1.164 |
| RECIST (version 1.1) response | 33 | 0.3743 | 0.682 | 0.293-1.586 |
| pEBV DNA CL < 8 days | 28 | 0.4798 | 0.638 | 0.184-2.217 |
| pEBV DNA CL < 10 days | 28 | 0.1545 | 0.504 | 0.196-1.294 |
| pEBV DNA CL < 15 days | 28 | 0.2779 | 0.600 | 0.239-1.510 |
| pEBV DNA CL <10 & >50% drop in sum of SUVmax | 28 | **0.0228** | 0.235 | 0.068-0.817 |
| pEBV DNA CL <15 & >50% drop in sum of SUVmax | 28 | 0.0530 | 0.332 | 0.109-1.014 |

(**Legend**: ECOG PS = eastern cooperative group performance status, SUVmax = maximal standard uptake value, CL = clearance, CI = confidence interval, pEBV DNA = plasma Epstein Barr virus DNA)
