# Supplementary material for: Prospective evaluation of plasma Epstein–Barr virus DNA clearance and fluorodeoxyglucose positron emission scan in assessing early response to chemotherapy in patients with advanced or recurrent nasopharyngeal carcinoma
Source: Br J Cancer. 2018 Mar 20;118(8):1051–5. doi: 10.1038/s41416-018-0026-9 (PMC5931094; doi:10.1038/s41416-018-0026-9)
Supplement: Supplementary file 5 — Supplementary Table 5 [file 41416_2018_26_MOESM5_ESM.docx]

**Supplemental Table 5: Progression-free survival – palliative subgroup (univariate analysis)**

| **Variable name** | **N** | **P-value** | **Hazard Ratio** | **95% C.I.** |
| --- | --- | --- | --- | --- |
| Advanced age | 33 | **0.0051** | 1.087 | 1.025-1.153 |
| Male gender | 33 | 0.1553 | 0.456 | 0.155-1.347 |
| ECOG performance (0 v.s. 1-2) | 33 | 0.1015 | 1.978 | 0.874-4.472 |
| >30% drop in sum of SUVmax | 33 | 0.0594 | 0.465 | 0.210-1.031 |
| >40% drop in sum of SUVmax | 33 | **0.0137** | 0.363 | 0.162-0.813 |
| >50% drop in sum of SUVmax | 33 | **0.0047** | 0.272 | 0.110-0.671 |
| RECIST 1.1 response | 33 | 0.3874 | 0.716 | 0.336-1.527 |
| pEBV DNA CL < 8 days | 28 | 0.1401 | 0.438 | 0.146-1.312 |
| pEBV DNA CL < 10 days | 28 | **0.0047** | 0.264 | 0.104-0.665 |
| pEBV DNA CL < 15 days | 28 | **0.0242** | 0.361 | 0.149-0.876 |
| pEBV DNA CL <10 & >50% drop in sum of SUVmax | 28 | **0.0042** | 0.191 | 0.062-0.593 |
| pEBV DNA CL <15 & >50% drop in sum of SUVmax | 28 | **0.0100** | 0.253 | 0.089-0.720 |

(**Legend**: ECOG PS = eastern cooperative group performance status, SUVmax = maximal standard uptake value, CL = clearance, CI = confidence interval, pEBV DNA = plasma Epstein Barr virus DNA)
