# Supplementary material for: Prospective evaluation of plasma Epstein–Barr virus DNA clearance and fluorodeoxyglucose positron emission scan in assessing early response to chemotherapy in patients with advanced or recurrent nasopharyngeal carcinoma
Source: Br J Cancer. 2018 Mar 20;118(8):1051–5. doi: 10.1038/s41416-018-0026-9 (PMC5931094; doi:10.1038/s41416-018-0026-9)
Supplement: Supplementary file 6 — Supplementary Table 6 [file 41416_2018_26_MOESM6_ESM.docx]

**Supplemental Table 6: Overall survival – Induction group (univariate analysis)**

| **Variable name** | **N** | **P-value** | **Hazard Ratio** | **95% C.I.** |
| --- | --- | --- | --- | --- |
| Advanced age | 25 | 0.7553 | 0.988 | 0.919-1.064 |
| Male gender | 25 | 0.6036 | 1.753 | 0.211-14.586 |
| ECOG performance (0 v.s. 1-2) | 25 | 0.6572 | 1.407 | 0.312-6.351 |
| >30% drop in sum of SUVmax | 25 | 0.7568 | 0.765 | 0.140-4.174 |
| >40% drop in sum of SUVmax | 25 | 0.4451 | 0.537 | 0.109-2.645 |
| >50% drop in sum of SUVmax | 25 | 0.1209 | 0.183 | 0.021-1.565 |
| RECIST (version 1.1) response | 25 | 0.3273 | 0.471 | 0.104-2.125 |
| pEBV DNA CL < 8 days | 22 | 0.1553 | 0.280 | 0.048-1.621 |
| pEBV DNA CL < 10 days | 22 | 0.1054 | 0.242 | 0.044-1.348 |
| pEBV DNA CL < 15 days | 22 | 0.0697 | 0.240 | 0.051-1.122 |
| pEBV DNA CL <10 & >50% drop in sum of SUVmax | 22 | 0.9958 | NR | NR |
| pEBV DNA CL <15 & >50% drop in sum of SUVmax | 22 | 0.9958 | NR | NR |

(**Legend**: ECOG PS = eastern cooperative group performance status, SUVmax = maximal standard uptake value, CL = clearance, CI = confidence interval, pEBV DNA = plasma Epstein Barr virus DNA, NR = not reached)
