# Supplementary material for: Prospective evaluation of plasma Epstein–Barr virus DNA clearance and fluorodeoxyglucose positron emission scan in assessing early response to chemotherapy in patients with advanced or recurrent nasopharyngeal carcinoma
Source: Br J Cancer. 2018 Mar 20;118(8):1051–5. doi: 10.1038/s41416-018-0026-9 (PMC5931094; doi:10.1038/s41416-018-0026-9)
Supplement: Supplementary file 7 — Supplementary Table 7 [file 41416_2018_26_MOESM7_ESM.docx]

**Supplemental Table 7: Progression-free survival – Induction group (univariate analysis)**

| **Variable name** | **N** | **P-value** | **Hazard Ratio** | **95% C.I.** |
| --- | --- | --- | --- | --- |
| Advanced age | 25 | 0.9302 | 0.997 | 0.935-1.063 |
| Male gender | 25 | 0.4266 | 2.349 | 0.286-19.274 |
| ECOG performance (0 v.s. 1-2) | 25 | 0.9197 | 0.934 | 0.248-3.515 |
| >30% drop in sum of SUVmax | 25 | 0.3663 | 2.070 | 0.427-10.039 |
| >40% drop in sum of SUVmax | 25 | 0.5184 | 1.582 | 0.393-6.360 |
| >50% drop in sum of SUVmax | 25 | 0.8419 | 0.863 | 0.204-3.658 |
| RECIST 1.1 response | 25 | 0.7231 | 0.778 | 0.193-3.125 |
| pEBV DNA CL < 8 days | 22 | 0.2110 | 0.352 | 0.068-1.809 |
| pEBV DNA CL < 10 days | 22 | **0.0510** | 0.191 | 0.036-1.007 |
| pEBV DNA CL < 15 days | 22 | 0.1553 | 0.334 | 0.074-1.516 |
| pEBV DNA CL <10 & >50% drop in sum of SUVmax | 22 | 0.1008 | 0.166 | 0.019-1.418 |
| pEBV DNA CL <15 & >50% drop in sum of SUVmax | 22 | 0.2743 | 0.398 | 0.076-2.076 |

(**Legend**: ECOG PS = eastern cooperative group performance status, SUVmax = maximal standard uptake value, CL = clearance, CI = confidence interval, pEBV DNA = plasma Epstein Barr virus DNA)
